# Determining the Optimal Conditions for the Production by Supercritical CO_2_ of Biodegradable PLGA Foams for the Controlled Release of Rutin as a Medical Treatment

**DOI:** 10.3390/polym13101645

**Published:** 2021-05-19

**Authors:** Diego Valor, Antonio Montes, Marilia Monteiro, Ignacio García-Casas, Clara Pereyra, Enrique Martínez de la Ossa

**Affiliations:** 1 Department of Chemical Engineering and Food Technology, Faculty of Sciences, University of Cadiz, International Excellence Agrifood Campus (CeiA3), Campus Universitario Río San Pedro, 11510 Puerto Real, Spain; diego.valor@uca.es (D.V.); ignacio.casas@uca.es (I.G.-C.); clara.pereyra@uca.es (C.P.); enrique.martinezdelaossa@uca.es (E.M.d.l.O.); 2 Technische Hochschule Nürnberg, Fakultät Verfahrenstechnik, Wassertorstr. 10, 90489 Nuremberg, Germany; monteiroma69365@th-nuernberg.de

**Keywords:** PLGA, supercritical CO_2_ foaming, rutin, scaffolds, release profile

## Abstract

Poly(D,L,-lactide-*co*-glycolide) (PLGA) foam samples impregnated with rutin were successfully produced by supercritical foaming processes. A number of parameters such as pressure (80–200 bar), temperature (35–55 °C), depressurization rate (5–100 bar/min), ratio lactide:glycolide of the poly(D,L,-lactide-*co*-glycolide) (50:50 and 75:25) were studied to determine their effect on the expansion factor and on the glass transition temperature of the polymer foams and their consequences on the release profile of the rutin entrapped in them. The impregnated foams were characterized by scanning electron microscopy, differential scanning calorimetry, and mercury intrusion porosimetry. A greater impregnation of rutin into the polymer foam pores was observed as pressure was increased. The release of rutin in a phosphate buffer solution was investigated. The controlled release tests confirmed that the modification of certain variables would result in considerable differences in the drug release profiles. Thus, five-day drug release periods were achieved under high pressure and temperature while the depressurization rate remained low.

## 1. Introduction

Supercritical carbon dioxide (scCO_2_) has proven to have a significant effect on many commercial polymers. This is why it is used in a large number of applications, such as the production of drug controlled release methods based on the use of impregnated scaffolds or the encapsulation of the active substances [1].

scCO_2_ has a plasticizing effect on polymers. This means that their melting point (T_m_), glass transition temperature (T_g_) and viscosity, among other properties, decrease. This effect, together with the fact that CO_2_ is a reusable and non-toxic element, makes of it an excellent option for the processing of polymers [1,2].

The usage and manufacturing of porous materials for medical purposes has experienced a considerable growth in recent years thanks to the intensive biomedical research that has been carried out. Some of the techniques that have been used until now for the production of scaffolds present some disadvantages, such as the usage of organic solvents [3,4]. Organic solvents can be toxic and harmful for the environment and their disposal can be rather costly. Other techniques that are also implemented, such as extrusion or compression molding [5,6,7], typically use high temperatures that can degrade some polymers. Moreover, if polymers need to be heated to get impregnated with bioactive compounds, which, in most cases, are thermolabile substances, such substances may get damaged because of the high processing temperatures [8]. In this respect, the use of supercritical CO_2_ blown into the system to produce the scaffolds presents some advantages, including the fact that it does not require the use of co-solvents and therefore, no high temperatures are applied to remove them from the polymer. This also makes of supercritical foaming an improved environmentally friendly process.

When a porous structure is created by supercritical CO_2_, the supercritical phase comes into contact with the polymer, which is plasticized because of its reduced melting point or glass transition temperature [9]. The system remains saturated over this contact time, and during the depressurization stage, the carbon dioxide supersaturation in the polymer matrix causes the nucleation and growth of the porous cells within the polymeric matrix [10]. In a supercritical foaming process, a small amount of CO_2_ dissolved into the polymers may bring down both the glass transition temperature (T_g_) and the polymer viscosity. As a result, bioactive compounds can be incorporated into the polymer at low temperatures. In order for the process to take place, it is necessary that the CO_2_ dissolves a sufficient quantity of the polymer. This fact makes supercritical foaming a suitable technology for production of foams of sensitive polymers such as amorphous and low molecular weight [11,12].

Among the biopolymers used in the development of microparticulate systems for controlled release, aliphatic polyesters, consisting of monomeric units of lactic acid and glycolic acid, are the most relevant ones. Poly(D,L,-lactide-*co*-glycolide) (PLGA) is an amorphous polyester with a glass transition temperature which varies depending on its lactide:glycolide ratio [13]. According to the literature, the T_g_ before the foaming process of PLGA 50:50 is 37 °C, while the T_g_ corresponding to PLGA 75:25 is 43 °C [14]. Many studies have investigated the effects of some of the variables that influence the process such as temperature, pressure, depressurization rate or the ratio lactide to glycolide of the polymer over the single stage supercritical foaming/impregnation process. According to many of these studies, the viability of the polymer to be impregnated by an active principle was demonstrated by the swelling that it experiences during the depressurization step.

PLGA has been used in locally implanted medical devices, including scaffolds for controlled drug release and to enhance drug bioavailability in tissue repairing processes [15]. The studies conducted have achieved the impregnation of gemcitabine in PLGA foams from ethyl lactate solutions of gemcitabine [16], mesoporous bioactive glass particles (MBGs) have been incorporated into PLGA [17], PLGA composite foams were produced using phosphate glass particles as filler [18], thymol has been impregnated into PLGA for controlled release [19] and bioactive lipids have been incorporated to PLGA scaffolds [20]. In some cases, such as bone tissue repair, the action of the drug is to be prolonged for as long as months. On the other hand, porosity and pore size must be taken into account so that they support the infiltration and proliferation of a variety of cells, including among others osteogenic cells [21]. PLGA implants can gradually release in situ an entrapped active substance as the scaffold is degraded. This allows a prolonged releasing period of the substance, that can exert its bioactivity throughout such controlled degradation time [22].

In contrast, Sun et al. explained that most of the kartogenin, a potential chondrogenesis promoter in cartilage tissue engineering—injected into articular cavities—is absorbed by the circulatory system. In this sense, PLGA scaffolds containing microspheres of kartogenin for its controlled delivery into articular cavities could be a suitable treatment [23].

Rutin is a natural flavonoid with significant scavenging properties on oxygen radicals in vivo and in vitro. It is a low molecular weight polyphenolic compound that has been extensively used in the treatment of different diseases because of its varied pharmacological properties, which includes antiallergic, anti-inflammatory, antibacterial, and antiviral activities [24]. Some authors have used supercritical antisolvent processes to precipitate or encapsulate rutin, either by itself or trapped inside another material [25,26]. However, supercritical solvent impregnation (SSI) has never been used for this purpose.

The aim of this work is to evaluate the use of a biodegradable PLGA polymeric foamy material to produce scaffolds to be used for the controlled release of rutin through functional implants. For this purpose, the authors have examined and evaluated a number of relevant variables, such as temperature (T), pressure (P), depressurization rate (Dr), and PLGA ratio lactide:glycolide and their influence on the polymer expansion factor, textural properties, and rutin loads and deliveries in order to determine the optimal conditions of the supercritical foaming/impregnation process.

## 2. Materials and Methods

### 2.1. Materials

Commercial poly (D,L,-lactide-*co*-glycolide) (lactide:glycolide 75:25) (molecular weight 76,000–115,000 g/mol), poly (D,L,-lactide-*co*-glycolide) (lactide:glycolide 50:50) (molecular weight 24,000–38,000 g/mol) and rutin was provided by Sigma–Aldrich (Madrid, Spain). Sodium-dihydrogen phosphate and sodium hydroxide were purchased from Panreac Applychem (Barcelona, Spain). CO_2_ with a minimum purity of 99.8% was supplied by Linde (Barcelona, Spain).

### 2.2. Foaming and Impregnation Process

**Figure 1:**
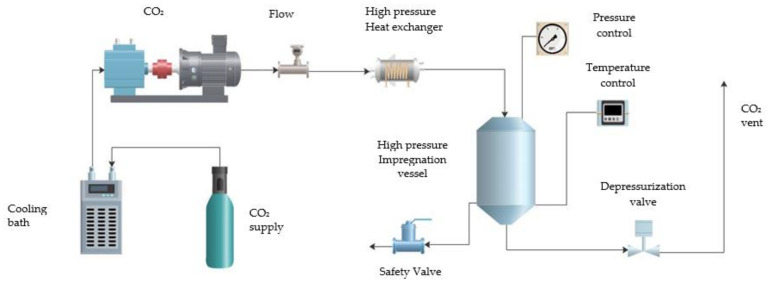
Diagram of the foaming/impregnation equipment setup.

The foaming/impregnation experiments were carried out in a pilot plant as shown in Figure 1. The setup included refrigeration system for high-pressure CO_2_ boosting (CO_2_ bottle, cooler, and high-pressure pump), a heat exchanger to adjust the temperature and a 257 mL vessel. Therefore, in order to foam the PLGA samples and impregnate them with rutin, initially, 0.2 g of each substance were placed for each run in a cylindrical aluminum foil support inside the vessel. The vessel was then filled with high-pressure CO_2_, which had been previously cooled and compressed by means of a high-pressure pump and pre-heated up to the desired conditions. This impregnation stage was held for 2 h in every case to make sure that the plasticization of the polymer took place. Then, the output valve was opened to let the CO_2_ out and depressurize the system.

A factorial design 2^4^ was performed in order to identify the main factors affecting the foaming and impregnation process of the PLGA polymer using rutin under supercritical conditions. A total of 18 experiments (with two central points) were carried out where ratio lactide:glycolide of the PLGA, pressure, temperature and depressurization rate were the modified variables. Each variable was set at two levels as shown in Table 1. The design was carried out by means of the software application Statgraphics Centurion 19.

### 2.3. Thermal Analysis

Differential scanning calorimeter (DSC Q100, TA Instruments, New Castle, USA) designed for the measurements of ambient temperature up to 220 °C was used to determine the thermal properties of the native polymers and the impregnated foams. The samples (5 mg) were heated up from room temperature (25 °C) until 220 °C according to a heating ramp of 10 °C/min, and finally heated up to 220 °C following the same ramp. The measurements began with a 2-min isothermal stage to even up the initial temperature. All the tests were carried out under a protective nitrogen atmosphere.

### 2.4. Mercury Intrusion Porosimetry

The textural properties of the samples that presented a higher foaming effect were determined by Mercury Intrusion Porosimetry (PoreMaster 60GT, Quantachrome Instruments, Florida, USA). The porosimetry measurements were conducted using an equilibration time of 10 s, the pressure in the vessel was increased to 50 psia and then gradually brought down to atmospheric pressure.

### 2.5. Scanning Electron Microscopy

The morphology of the foamed polymer samples was observed by Scanning Electron Microscope (SEM). A Nova NanoSEM 450^TM^ scanning electron microscope (Elecmi, Zaragoza, Spain) with an accelerating voltage of 30kV was used. Crossed sections of the polymer and their front side were selected for their observation. Previous to their analysis, the samples were coated with a 10 nm thick gold layer in order to improve their conductivity.

### 2.6. In Vitro Release Test

In order to determine the rutin’s release kinetics, rutin-impregnated PLGA foam samples were weighed (40 mg) and suspended in 25 mL of 0.05 M phosphate buffer solution (PBS) stirred at 200 rpm at constant 37 °C. The PBS (1L) was prepared mixing 18.4 mL of monobasic potassium phosphate with 31.6 mL of dibasic potassium phosphate in distilled water at pH 6.8. Based on the calculations reported by Zhu et al. [27], the release of rutin into the solution was measured after 5, 15, 30, and 60 min, and then every hour by determining its concentration based on an aliquot (3 mL) of the release solution measured by means of a UV–vis spectrophotometer (Shimadzu, Columbia, USA) at λ = 360 nm.

## 3. Results and Discussion

### 3.1. Foaming and Impregnation Runs

**Figure 2:**
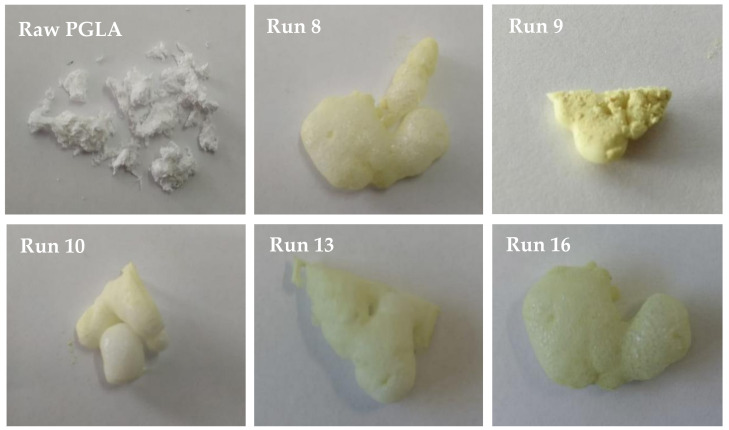
Photos of the raw PGLA and the resulting samples from runs 8, 9, 10, 13, and 16.

According to their acquired homogenous color, most of the foamed PLGA polymers that were obtained presented a homogeneous impregnation with rutin. However, part of the injected rutin was did not impregnate the foam and was collected back. This was probably due to the low solubility of rutin in supercritical CO_2_ [28,29]. The experimental design and the observed responses are presented in Table 2. The density of CO_2_ is shown for each of the conditions under which the experiments were carried out. The expansion factor was calculated as the ratio between the final volume of polymer foam samples and their initial volume (V_f_/V_0_). This would be considered as a reference of the effectiveness of the foaming process. Runs 2, 8, 10, 11, 13, and 16—which were generally conducted under high pressure (140 and 200 bar) and a greater depressurization rate—presented a higher expansion factor corresponding to a greater foaming effect (Table 2). The foams from runs 1, 4, 9, and 12 were calculated lower expansion factors, which was evidenced by a poorer foaming effect, with smaller size bubble clusters. Most of these less successful experiments had been carried out by depressurizing at a low rate (5 bar/min), which seems to indicate that the CO_2_ exhaust rate from the vessel could be a crucial factor with regard to the final foam structure. When low depressurization rates are applied, diffusion of part of the dissolved CO_2_ from the plasticized polymer into the vessel could be expected, directly affecting the gas concentration within the saturated polymer. As a result of the different CO_2_ concentrations in the polymer, the performance of the foaming process changes, mainly affecting pore nucleation and consequent polymer expansion. Previous works in the literature obtained a larger cell diameter and expansion using slower Dr [30], however, in the present study the opposite trend was observed. Figure 2 shows pictures of some of the experiments with the highest expansion factor or foaming effect, as well as the PLGA before being treated in order to compare their appearance. Run 9 is also shown as an example of the appearance of the experiments in which a lower expansion factor was obtained in the process.

**Figure 3:**
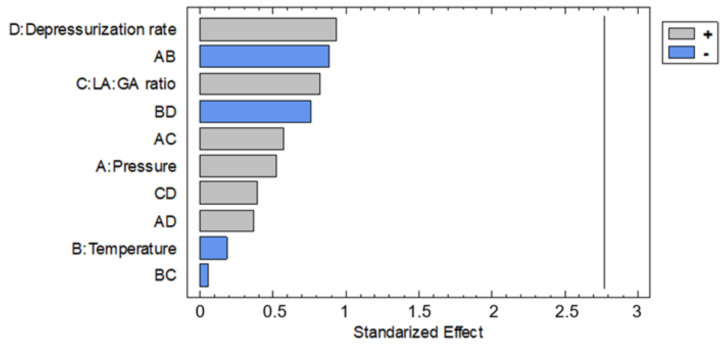
Effect of the considered variables on the expansion factor of the foamed polymer samples.

**Figure 4:**
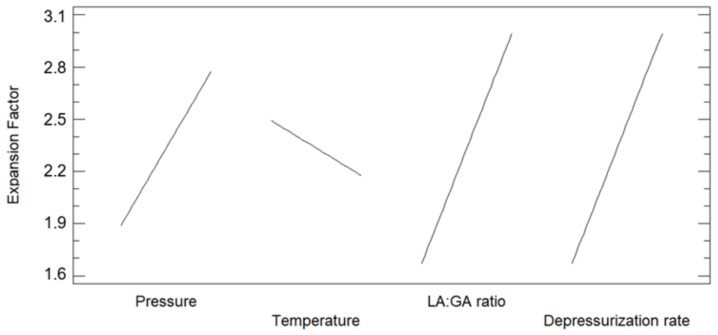
Main effects of the considered variables on the expansion factor of the foamed polymer samples.

Once analyzed, the results of design it can be observed in Figure 3 that Dr followed by pressure × temperature and LA/GA ratio of polymer had the highest level of significance on the expansion factor as it has been preliminary discussed. On the other hand, temperature and temperature × ratio effects had the lowest importance. In this way, the main effects on the expansion factor were determined by means of the design and presented in Figure 4. Thus, it was found out that the expansion factor increases with pressure, and decreases with temperature. The combination of pressure with temperature effect had high level of significance. In other words, expansion factor increases as CO_2_ density is greater (Table 2), which in turn means an increment of the solvent volume and accordingly its capacity to plasticize the polymer to enhance the foaming effect. On the other hand, the expansion factor increases as Dr increases, which confirms the results from preliminary analyses. Moreover, when the glycolic content is increased the expansion factor also increases. It seems that removing an extra methyl group produces lower steric hindrance and then increases the interaction between the carbonyl group and the CO_2_ molecules Thus, solubility and foaming effect is more pronounced due to scCO_2_ would plasticize the polymer easily. However other authors found out that increasing the lactic acid content in PLGA polymers increasing the solubility of CO_2_ in it due to the available free volume in the matrix is increased because of steric effects related with this extra methyl group and this fact is a more dominant factor than the steric hindrance [31].

**Figure 5:**
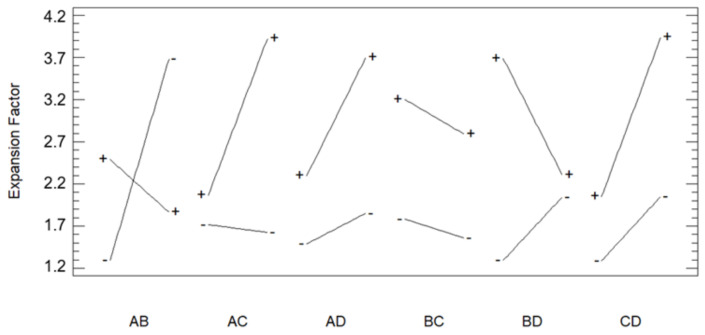
Effect from the interactions between the considered variables on the expansion factor of the foamed polymer samples (A: Pressure; B: Temperature; C: LA/GA ratio; D: Depressurization rate).

The degree of interaction between the different variables was also investigated (Figure 5) by analyzing their interaction plots. A significant interaction was observed between pressure and temperature. Thus, at low pressure the expansion factor increased when the temperature was increased. When the pressure increases, the opposite effect occurs, since the expansion factor increases as the temperature decreases. This may be due to the fact that CO_2_ has a higher density and greater penetration capacity at these conditions of pressure and temperature. A minor interaction between pressure and Dr was also observed, where the expansion factor would increase when a greater Dr was applied particularly at the higher values of the pressure range employed. Temperature also presented a notable interaction with Dr, which meant that the expansion factor would increase at low temperature levels when the Dr was also increased. On the other hand, the polymer ratio had a negligible interaction with temperature but a notable one with pressure, since the reduction in lactic content produced an improved foaming effect when under high pressure.

**Figure 6:**
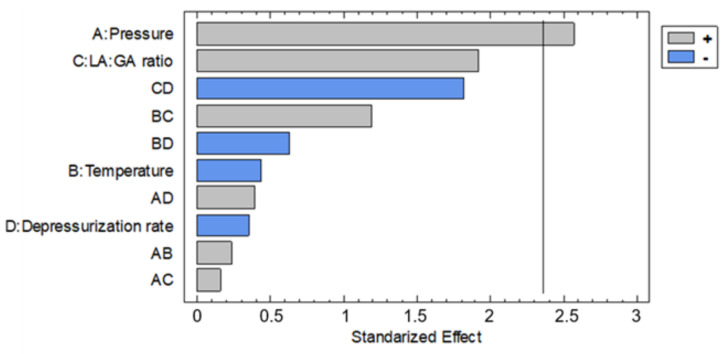
Effect of the considered variables on the rutin loads impregnated onto the foamed polymer samples.

**Figure 7:**
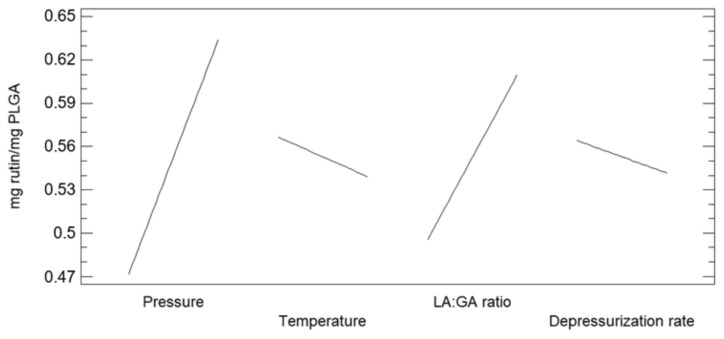
Main effects of the considered variables on the rutin loads impregnated onto the foamed polymer samples.

The amount of rutin loaded into the PLGA foams were calculated as the difference between the initial and the residual rutin that did not remain impregnated into the foam (Table 2). The greatest rutin loads were obtained from the experiments 2, 10, and 12, which had been carried out at the maximum pressure values of the range considered. Conversely, the smallest rutin loads were registered for the experiments 13 and 15, both of which had been carried out at low pressure. However, runs 1, 3, and 14, which had been conducted at low pressure levels had reached considerable rutin loads, demonstrated the effect of the interaction between certain parameters. Pressure, ratio, Dr × ratio and temperature × ratio were the more significant effects as can be seen in Figure 6. The main effects according to the design were investigated (Figure 7). Thus, it was observed that the greatest rutin loads were obtained at high pressure and low temperature. Since CO_2_ density is higher under these conditions, a greater amount of rutin was solved and available to be impregnated onto the foamed polymer. It should also be noted that rutin loads were also slightly higher when both Dr and lactic content values were lower.

**Figure 8:**
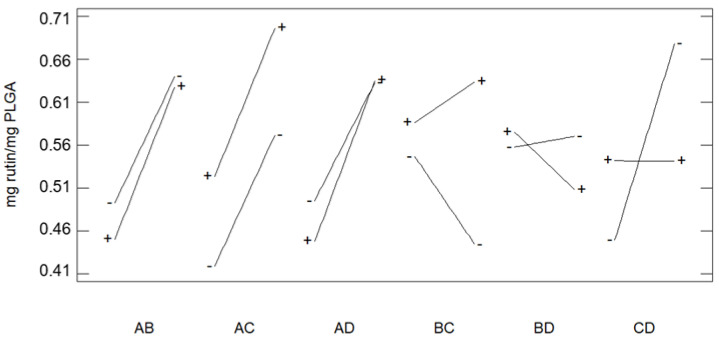
Effect of the interactions between the considered variables on the rutin loads impregnated onto the foamed polymer samples (A: Pressure; B: Temperature; C: LA/GA ratio; D: Depressurization rate).

The effect of the interactions between the different variables and rutin loads are shown in Figure 8. No relevant variations with regard to rutin load could be determined between pressure and temperature when the temperature remained constantly low while the pressure varied within the range considered for the experiments. Such minor variation is in accordance with the existing correlation between decreasing temperature and increasing CO_2_ density. On the other hand, Dr presented a notable inverse interaction with temperature and a moderate one with pressure, so that greater loads were achieved with lower Dr at low pressure and high temperature. Finally, the polymer ratio did not present any relevant interactions neither with pressure nor with temperature. However, a strong interaction was found with Dr, and expansion factor goes up when the glycolic content is increased at low Dr, and the opposite effect at high Dr.

### 3.2. Thermal Analysis

**Figure 9:**
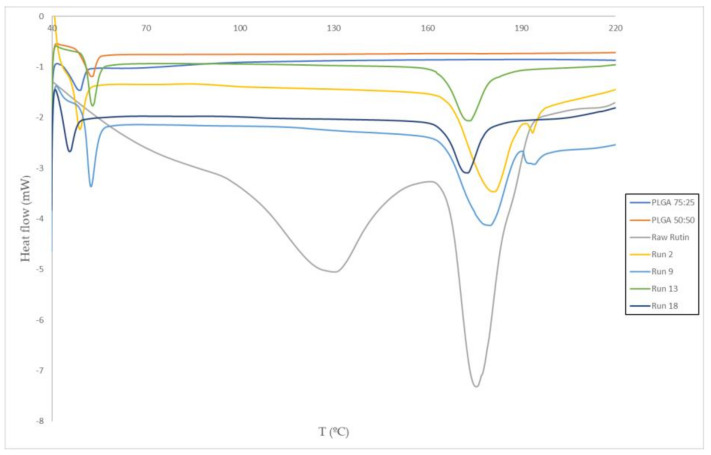
DSC tests of runs 2, 9, 13, 18, PLGA 50:50, PLGA 75:25, and raw rutin.

Figure 9 displays the DSC analyses of the most representative samples, with the two peaks corresponding to T_g_ and T_m_ (see all the DSC analyses in the Supplementary File). Glass transition temperatures of PLGA 50:50 (48.3 °C) and PLGA 75:25 (52.8 °C) were also measured prior to the supercritical foaming/impregnation process. The DSC analysis of raw rutin showed two typical bands, the first one at around 130 °C (characteristic of hydrated rutin) and the second one that is related to the melting point of rutin at around 176 °C in agreement with the literature [28]. In the impregnation experiments, the peak at 130 °C does not appear so there were produced foams with anhydrous rutin which a priori has a higher commercial value than rutin hydrate because of its higher antioxidant and biological activities per gram of compound [32,33,34]. Furthermore, in some cases the melting point of rutin decreases with respect to the raw rutin, as can be observed in Figure 9, due to its interaction with CO_2_ in supercritical phase. When a supercritical fluid is dissolved in an active compound the melting point of solid substances can be depressed considerably [35].

In contrast, it was observed that the T_g_ of the foamed polymer impregnated with rutin did not change significantly throughout the overall design, with a standard T_g_ deviation of 1.1 and 0.6 for the 75:25 and 50:50 PLGA experiments, respectively. Changes in glass transition temperature can lead to modifications in the properties of solids, which can significantly affect their application in the pharmaceutical field [36]. In any case, the T_g_ values obtained were always above human body temperature (37 °C) and this confirms that the produced foamed polymers are suitable for use as a drug delivery system in the pharmaceutical sector.

### 3.3. Scanning Electron Microscopy (SEM)

**Figure 10:**
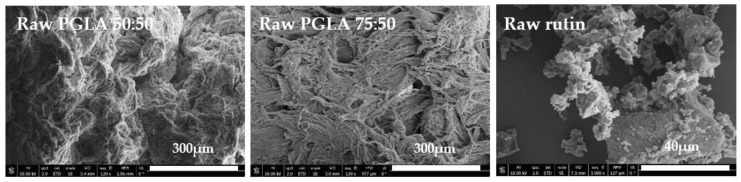
SEM images of raw PGLA and raw rutin.

**Figure 11:**
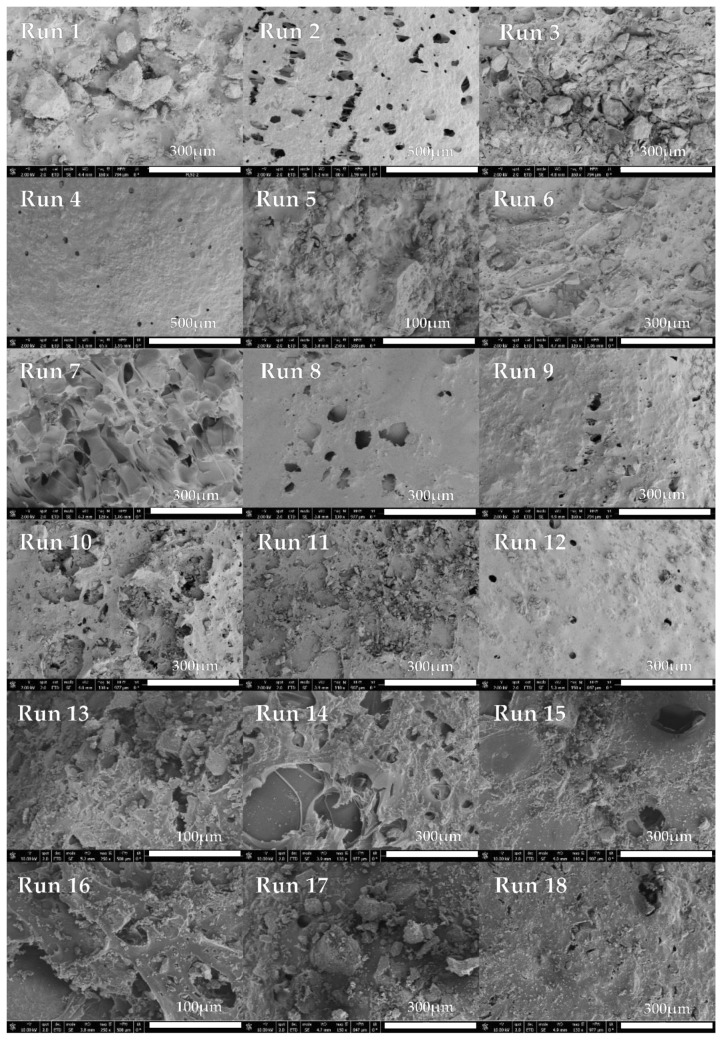
SEM images of foams processed.

In order to compare the results, the untreated compounds (Figure 10) and the foams obtained after the foaming/impregnation process (Figure 11) were analyzed by scanning electron microscopy. Both raw PLGA LA:GA ratios showed a heterogeneous structure with no pores present on the surface. On the other hand, most of the images of the foams that were obtained from the experiments show the formation of irregular porous structures, with clear differences according to the operating conditions used for their production. These heterogeneous structures could be due to the fact that rutin crystals act as foaming nuclei [31]. Some structural differences were observed as a function of pressure.

The appearance of pores on the polymer surface can be observed in runs 2, 4, 8, 9, 10, 12 (Figure 11) while in the other runs the pores are less apparent on the SEM images; all these runs were processed using high (200 bar) or medium pressure (140 bar). None of the runs conducted at low pressure (80 bar) resulted in the formation of a PLGA porous structure. This can be explained by the fact that, as the pressure increases, the solubility of CO_2_ increases, leading to a greater dissolution of the polymer in the supercritical phase, which results in a higher foam cell density as well as a smaller cell diameter [33,34]. As for the effect of temperature, in the aforementioned experiments in which a porous structure was generated, it was observed that with increasing temperature (55 °C) the pore size decreases. Furthermore, at higher temperature, the energy required for nucleation also increases, so a reduction in cell density also occurs [10].

### 3.4. Mercury Intrusion Porosimetry

**Figure 12:**
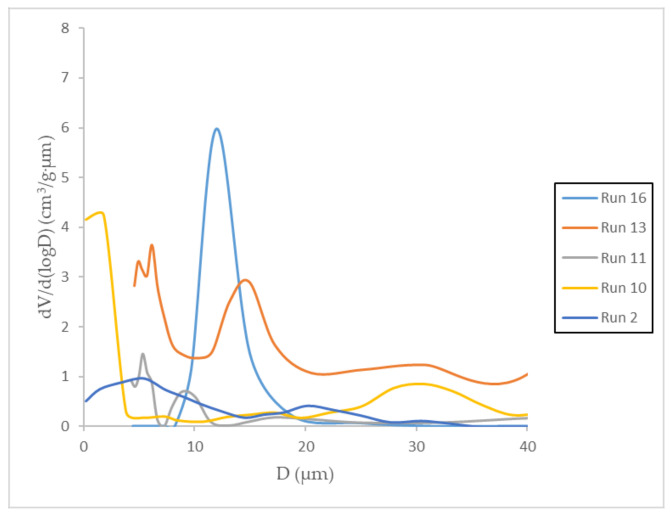
Pore size distribution in processed foams.

**Figure 13:**
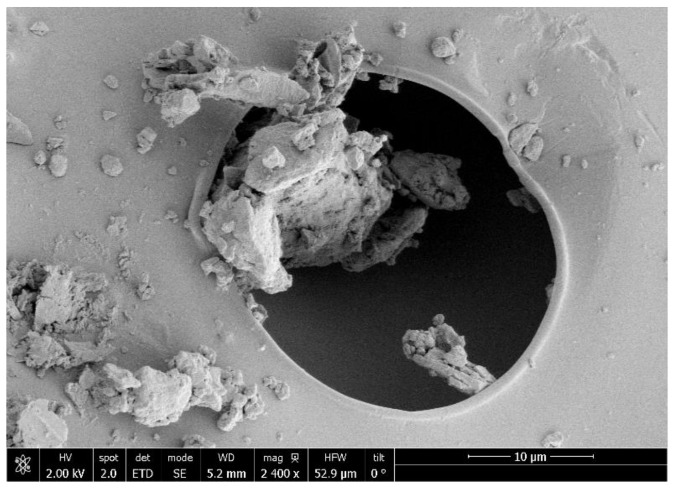
Rutin trapped in one of the pores of the foam obtained (Run 12).

The measurement of the volume of mercury intruding into a sample as a function of the increasing pressure allows pore size and volume distributions to be determined according to the pores that are accessible from the outside of a material. Porosimetry was performed on the samples with the highest expansion factor and their textural properties were collected in Table 3. It should be taken into account that in the process of depressurization, the effect of adiabatic cooling of the carbon dioxide is present inside the vessel especially in the case of high depressurization rates. During such cooling, the transition of carbon dioxide from the supercritical to subcritical state can occur. This transition can cause such local effects as occurrence of coexisting liquid and gaseous phases of carbon dioxide in the expanding pores and, sometimes, their transitions from an expansion mode to collapse. Pore size distribution is shown in Figure 12. All the analyzed samples had a similar pore volume, except for sample 10, where the pore volume was three times larger. This volume was divided into several pore size groups, being the smallest (1.80 µm) and the largest (30.66 µm). Run 13 had the largest surface area at five times larger than the rest. However, the pore size distribution was more heterogeneous, with three run groups averaging in pore size. The smallest pores were obtained in run 11 with two groups of pores sized between 5.32 and 9.04 microns. Run 16 was the experiment with the most uniform pore size, with a mean pore diameter of 11.97 microns. In any case, it is difficult to establish a correlation between the SEM images and the porosimetry data, among other things, because of the rutin that could be found on the surface of the polymer near and inside the pores. Figure 13 shows experiment 12 at higher magnification as an example of how rutin is deposited in the pores of the obtained polymer.

### 3.5. In Vitro Release Test

It should be noted that rutin has a low solubility in water (125 mg/L) and, therefore, by integrating it into the polymer foam, it should remain there for a long period of time [37]. Moreover, the acid–base equilibrium of biologically active rutin plays an important role which would result in different protonation degrees associated to their potential to transport the drug through biological membranes, which in turn would affect drug administration, distribution, metabolism, and excretion [38].

The in vitro process of rutin release from foams in a PBS solution (phosphate buffer to simulate intestinal fluid with pH 6.8) was studied. The rate of the drug release proved to be dependent on the solubility of rutin in the medium as well as on the morphology of the foams that had been obtained, since it has an influence on the way the polymer is eroded and diluted in the fluid [39]. The release process is also affected by the degradation and diffusion of the polymer through the matrix where the drug is distributed [16]. These characteristics will be affected by variations in pressure, temperature, depressurization rate, or polymer LA:GA ratio. Therefore, the degree of influence exerted by these variables should be determined.

**Figure 14:**
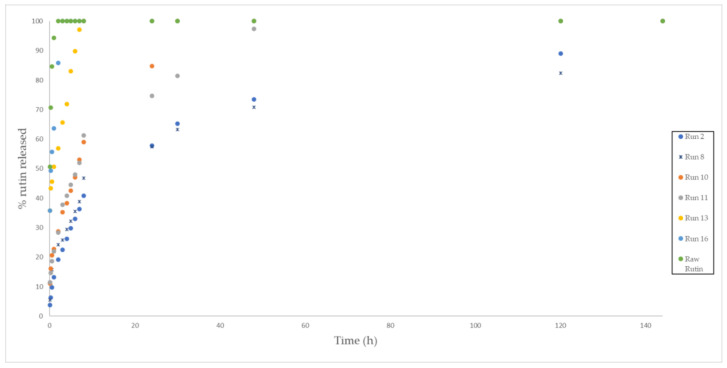
Rutin release profiles from the polymer foams (Runs 2, 10, 11, 13, 16, and raw rutin) into the PBS solution.

**Figure 15:**
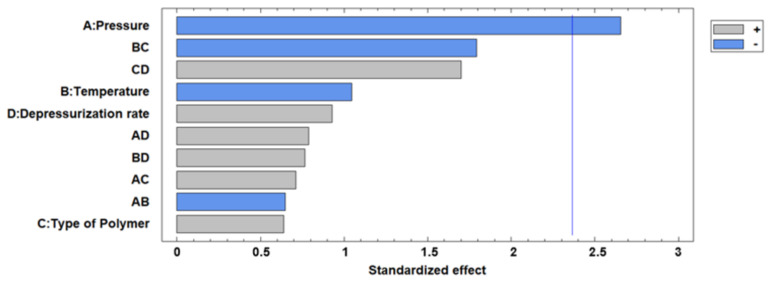
Standardized pareto diagram corresponding to the percentage of rutin released into the PBS solution after 8 h.

In general, an increase in pressure and temperature resulted in an impregnated foamed polymer where the time required for the rutin to be completely released into the solution would increase until a maximum of 120 h. This could be explained by the improved penetration of the supercritical CO_2_ that results in an increasing amount of rutin impregnated inside the polymer. Figure 14 shows the % of rutin released for up to 5 days into the PBS solutions by the impregnated foams obtained in experiments 2, 10, 11, 13, and 16, compared to the percentage of dissolution of raw rutin. These experiments were selected since they were, in general, the ones with the highest foam expansion factor. It can be seen that runs 2, 10, and 11 showed a delayed release, with run 2 reaching a release profile of 120 h. All of these foams had been produced at high pressure and presented a pore volume and expansion factor according to the previous descriptions (Table 2 and Table 3). The foam samples from experiment 16, despite being produced at high pressure, exhibited a faster release of rutin compared to the other foams obtained at the same pressure. This could be explained by the impregnation of the rutin on the surface of the polymer rather than inside the foam pores. It is also important to note the effect of the porosity or cell structure of the obtained foams on the obtained release data. Comparing the specific surface area data of runs 2 and 13 (Table 3), which show appreciable differences in the speed of rutin release, seems to indicate that a decrease in surface area benefits the entrapment of the drug within the polymer rather than being superficially impregnated, thus enhancing or delaying release into the PBS solution. The Pareto diagram of the rutin releasing profiles can be seen in Figure 15. A correlation between the effect of pressure and temperature on the expansion factor was observed in the same terms on the releasing of the rutin—i.e., the higher the pressure and the lower the temperature—the longer the releasing time. However, depressurization rate showed the opposite trend, i.e., the lower the Dr, the more slowly the rutin was released. As mentioned above, at high Dr levels, pore collapse can occur due to the adiabatic cooling of the CO_2_, forcing the drug to remain on the surface of the foams and facilitating its rapid dissolution and release.

**Figure 16:**
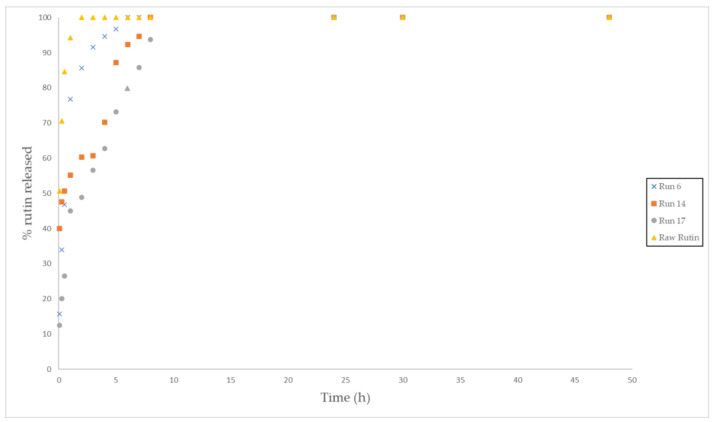
Rutin release profiles from the polymer foams (Runs 6, 14, 17, and raw rutin) into the PBS solution.

In general, low pressures resulted in foams that released the rutin in a reduced time. Figure 16 shows the percentage of rutin released by the foam obtained from experiments 6, 14, 17, which had been carried out at 80 bar, and raw rutin. The release profile of these compound systems where the rutin was fully released in 10 h is clearly different from that shown by those displayed in Figure 14. The rest of the release profiles of the different samples are available in the Supplementary Material. All of this corroborates that experiment 2, performed at high pressure, high temperature and low Dr (200 bar, 55 °C and 5 bar/min) displayed the longest releasing time (being the one with the largest rutin loading). It is then confirmed how important it is to control the variables during the foaming process. In fact, some of the foamed polymers that had been produced were capable of retaining the drug for up to 5 days, while others released the drug completely after 10 h. It should be noted that for certain biomedical applications such as implants in tissue regeneration, a slow and prolonged administration of certain drugs is crucial if the desired therapeutic effect is to be achieved.

## 4. Conclusions

The foaming and impregnation of PLGA polymer with rutin by means of supercritical CO_2_ was successfully achieved. The influence of specific process variables such as pressure, temperature, depressurization rate, and lactide:glycolide ratio on the resulting foam structure, its expansion factor, polymer’s glass transition temperature and rutin release profile were determined. The polymer’s glass transition temperature did not change significantly with its processing. The experiments at high pressure and lower temperature obtained a greater foaming effect, which has been proved to significantly influence the responses of interest. A porosimetry study was carried out on those polymer foams that had displayed, in general, a higher expansion factor, with pore sizes from 1.80 up to 30.66 mm. The PLGA/rutin combined systems produced with the largest rutin load and longest drug releasing profile were obtained at the highest pressures and lowest depressurization rates assayed. The analysis of the releasing of the drug into the PBS solution (neutral pH) revealed noticeable differences that ranged from 10 h in the case of the foams produced at low pressure and as much as 5 days for those produced under the highest pressure values. Based on the release profiles obtained, the efficacy of PLGA as a carrier agent for the controlled release of rutin to be administered for biomedical purposes has been demonstrated.

## Figures and Tables

**Table 1:** Different factors and levels considered for the impregnation experiments

Factors	Level 1	Level 2
Ratio lactide:glycolide (LA:GA)	75:25	50:50
Pressure (bar)	80	200
Temperature (°C)	35	55
Depressurization rate (bar/min)	5	100

**Table 2:** Experimental design and observed responses.

Runs	P (bar)	T (°C)	Dr (bar/min)	Ratio LA:GA	CO_2_ Density (kg/m^3^)	Expansion Factor (V_f_/V_0_) ^1^	mg Rutin Loaded/ mg PLGA
1	80	55	5	50:50	103.12	1	0.63
2	200	55	5	50:50	753.71	2.60	0.75
3	80	35	100	75:25	490.62	1.63	0.69
4	200	35	5	75:25	865.65	1.52	0.57
5	200	55	100	75:25	753.71	1	0.59
6	80	35	100	50:50	490.62	1.11	0.37
7	140	45	50	75:25	709.66	1	0.39
8	140	45	50	50:50	709.66	5.60	0.48
9	200	55	5	75:25	753.71	1.42	0.52
10	200	55	100	50:50	753.71	2.03	0.71
11	200	35	100	75:25	865.65	2.81	0.66
12	200	35	5	50:50	865.65	1.22	0.75
13	80	55	100	75:25	103.12	3.02	0.28
14	80	35	5	50:50	490.62	1	0.65
15	80	35	5	75:25	490.62	1.17	0.32
16	200	35	100	50:50	865.65	8.55	0.64
17	80	55	5	75:25	103.12	1.05	0.44
18	80	55	100	50:50	103.12	1	0.51

^1^ V_f_/V_0_ = foamed polymer to the raw polymer volume ratio.

**Table 3:** Textural properties of the foamed polymer samples.

Runs	Vp	S	Mean Dp (μm)		
	(cm^3^/g)	(m^2^/g)	Peak1	Peak2	Peak3
2	1.37	0.11	5.48	20.17	----
10	6.64	0.25	1.80	30.66	----
11	1.95	0.22	5.32	9.04	----
13	1.25	1.17	5.26	6.15	14.93
16	0.84	0.27	11.97	----	----

## Supplementary Materials

The following are available online at https://www.mdpi.com/article/10.3390/polym13101645/s1. DSC tests of experiments 1–18, raw PLGA and rutin. Release profile of experiments 1–18 and raw rutin.
