# Supplementary material for: Determining the Optimal Conditions for the Production by Supercritical CO2 of Biodegradable PLGA Foams for the Controlled Release of Rutin as a Medical Treatment
Source: Polymers (Basel). 2021 May 19;13(10):1645. doi: 10.3390/polym13101645 (PMC8158779; doi:10.3390/polym13101645)

Figure 3. Effect of the considered variables on the expansion factor of the foamed polymer samples.

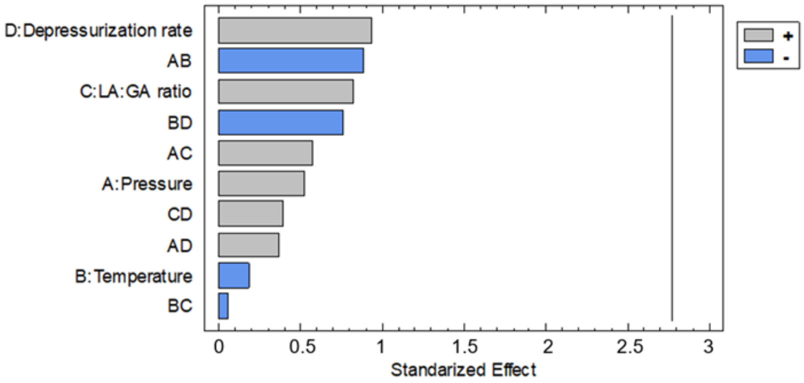

Supplement: Supplementary file 1 [file polymers-13-01645-s001.zip › Figure 3.pdf]
