# Supplementary figures and images for: Determining the Optimal Conditions for the Production by Supercritical CO2 of Biodegradable PLGA Foams for the Controlled Release of Rutin as a Medical Treatment
Source: Polymers (Basel). 2021 May 19;13(10):1645. doi: 10.3390/polym13101645 (PMC8158779; doi:10.3390/polym13101645)

**Figure 9.** DSC tests of runs 2, 9, 13, 18, PLGA 50:50, PLGA 75:25 and raw rutin.

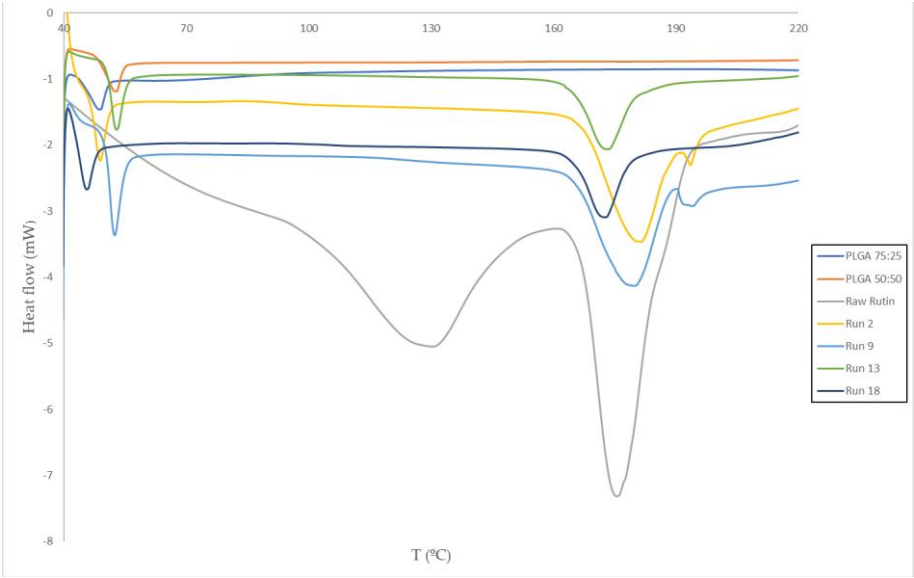

Supplement: Supplementary file 1 [file polymers-13-01645-s001.zip › Figure 9.pdf]

**Figure 10.** SEM images of raw PGLA and raw rutin.

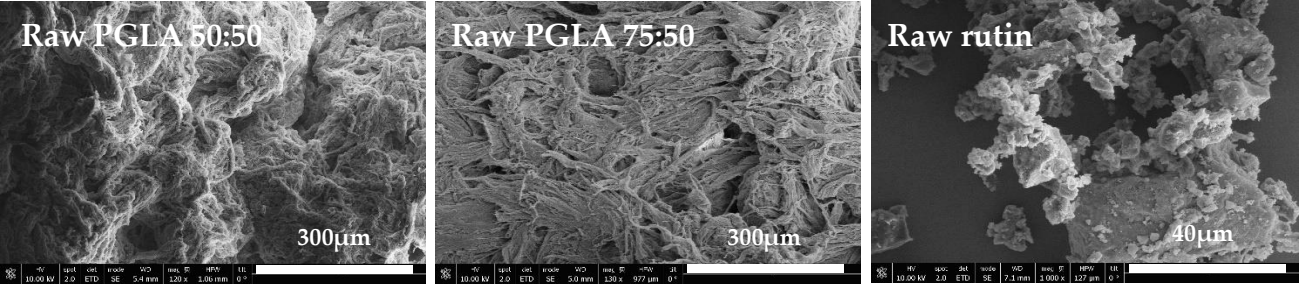

Supplement: Supplementary file 1 [file polymers-13-01645-s001.zip › Figure 10.pdf]

Figure 11. SEM images of foams processed.

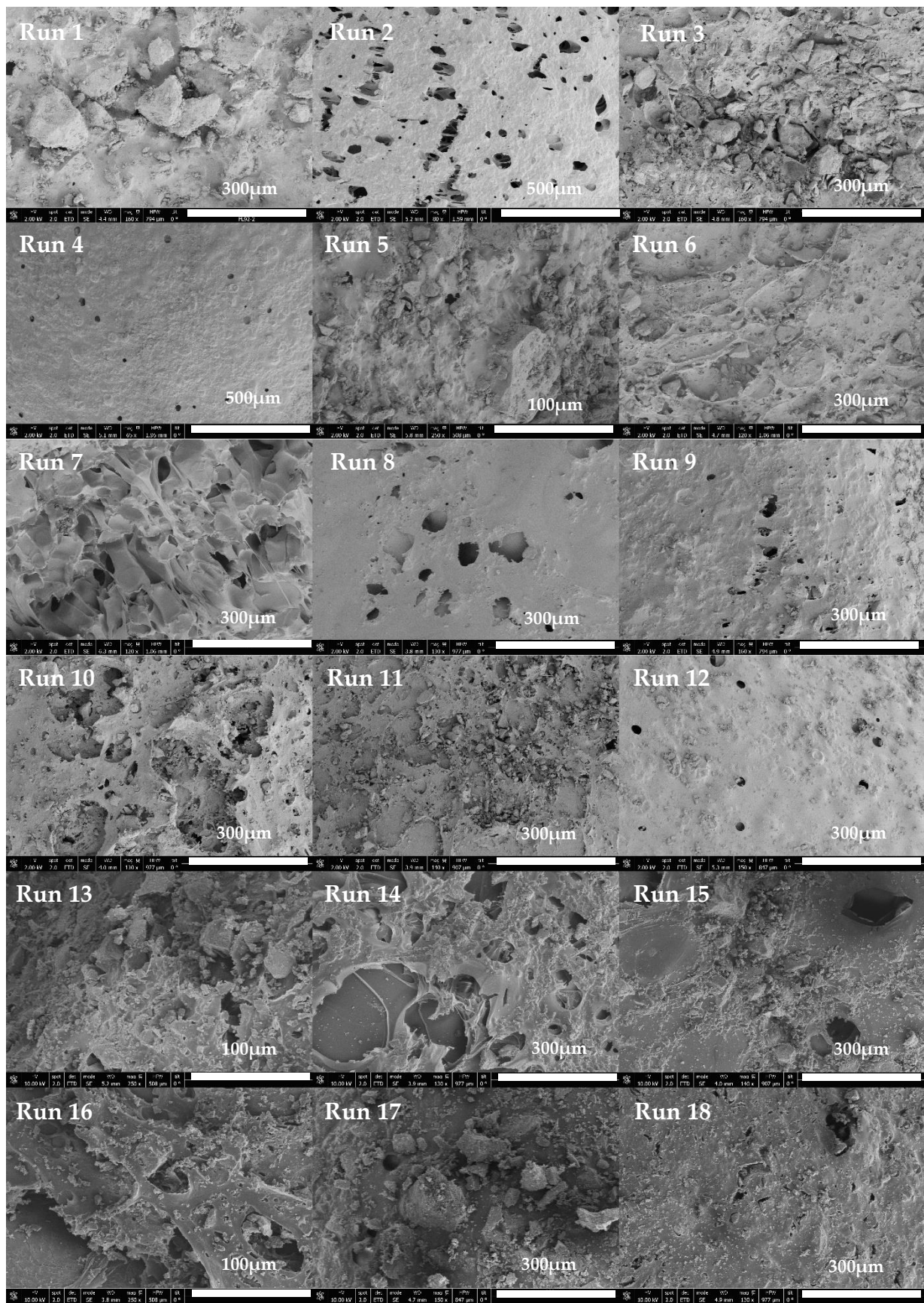

Supplement: Supplementary file 1 [file polymers-13-01645-s001.zip › Figure 11.pdf]

**Figure 12.** Pore size distribution in processed foams.

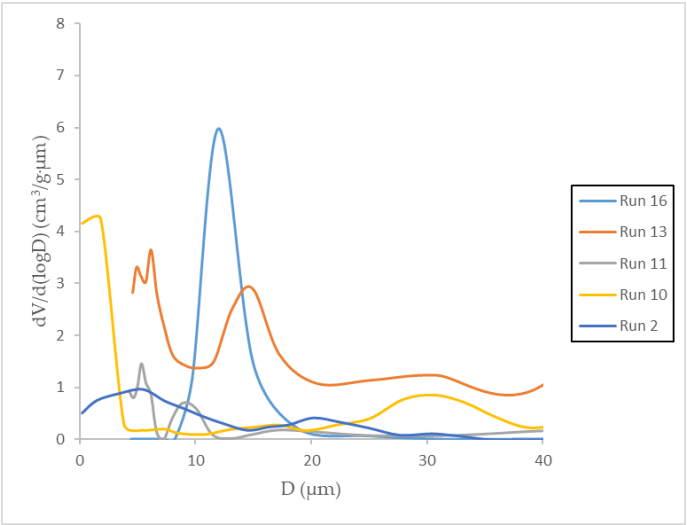

Supplement: Supplementary file 1 [file polymers-13-01645-s001.zip › Figure 12.pdf]

**Figure 13.** Rutin trapped in one of the pores of the foam obtained (Run 12).

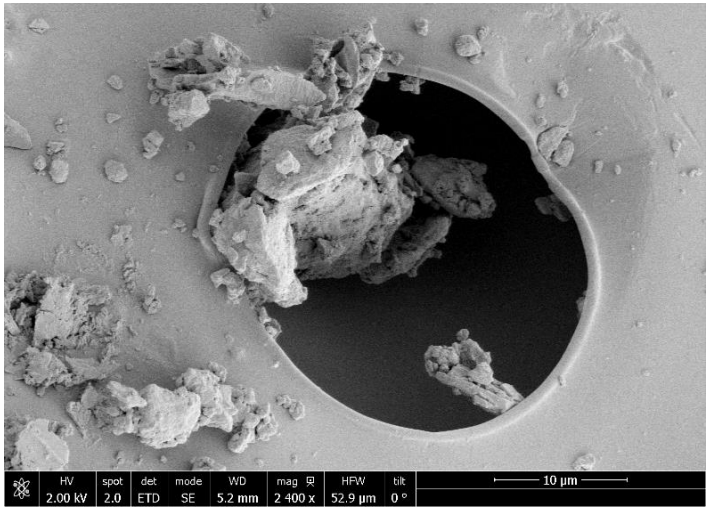

Supplement: Supplementary file 1 [file polymers-13-01645-s001.zip › Figure 13.pdf]

**Figure 14.** Rutin release profiles from the polymer foams (Runs 2,10,11,13,16 and raw rutin) into the PBS solution.

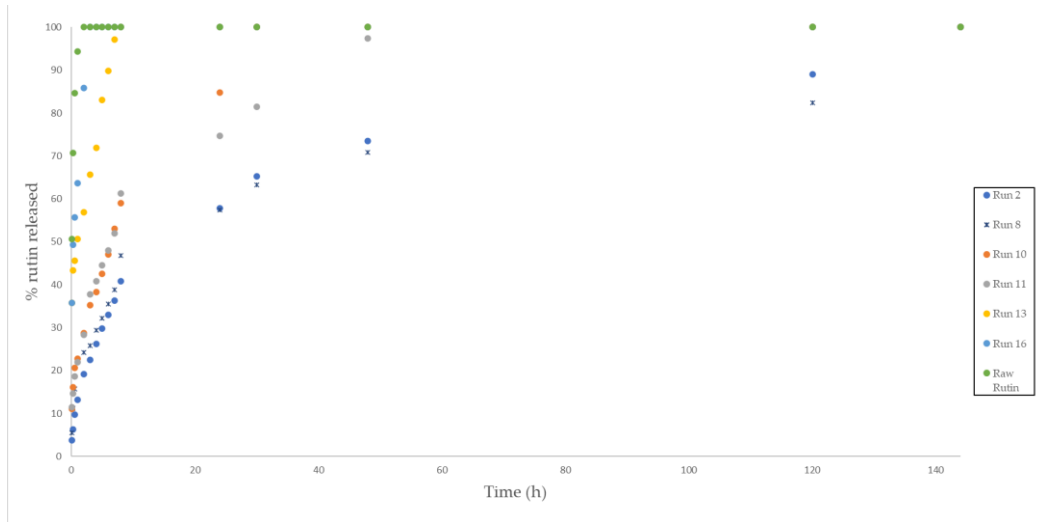

Supplement: Supplementary file 1 [file polymers-13-01645-s001.zip › Figure 14.pdf]

**Figure 16.** Rutin release profiles from the polymer foams (Runs 6,14,17 and raw rutin) into the PBS solution.

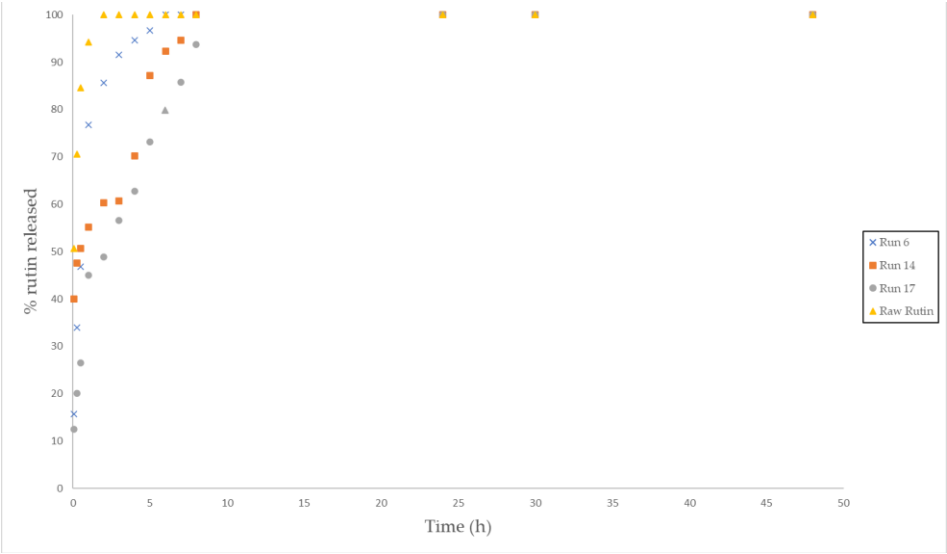

Supplement: Supplementary file 1 [file polymers-13-01645-s001.zip › Figure 16.pdf]

**Figure 1.** Diagram of the foaming/impregnation equipment setup.

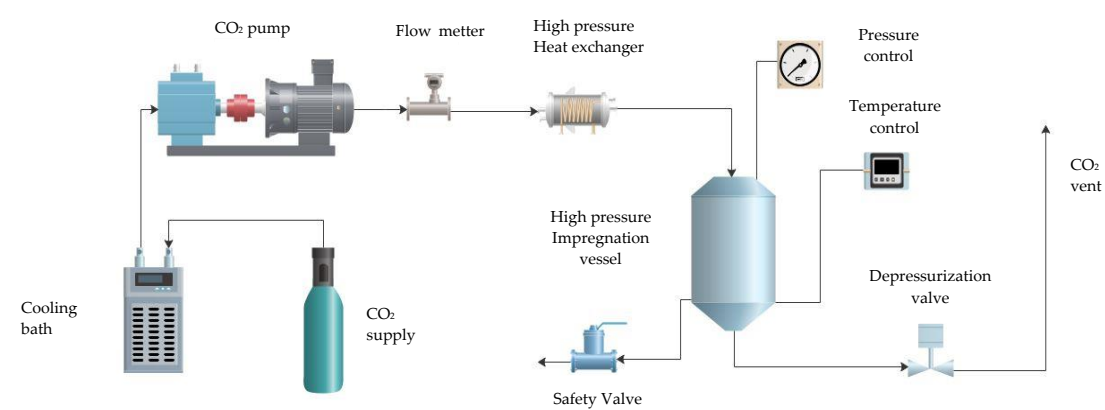

Supplement: Supplementary file 1 [file polymers-13-01645-s001.zip › Figure 1.pdf]

**Figure 2.** Photos of the raw PGLA and the resulting samples from runs 8, 9,10,13 and 16.

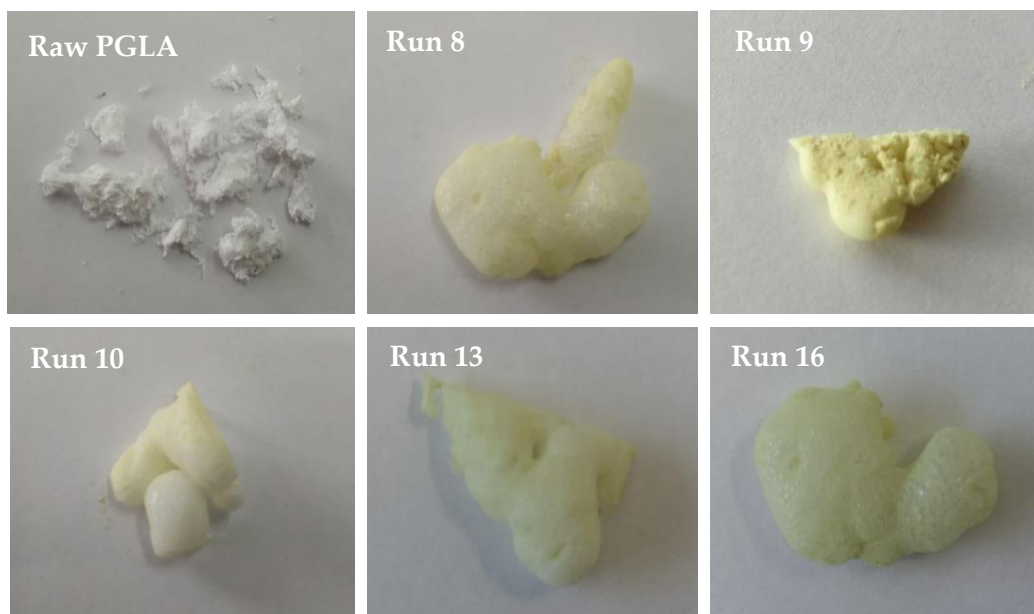

Supplement: Supplementary file 1 [file polymers-13-01645-s001.zip › Figure 2.pdf]
